# Supplementary figures and images for: Dynamics and variability in the pleiotropic effects of adaptation in laboratory budding yeast populations
Source: eLife. 2021 Oct 1;10:e70918. doi: 10.7554/eLife.70918 (PMC8579951; doi:10.7554/eLife.70918)

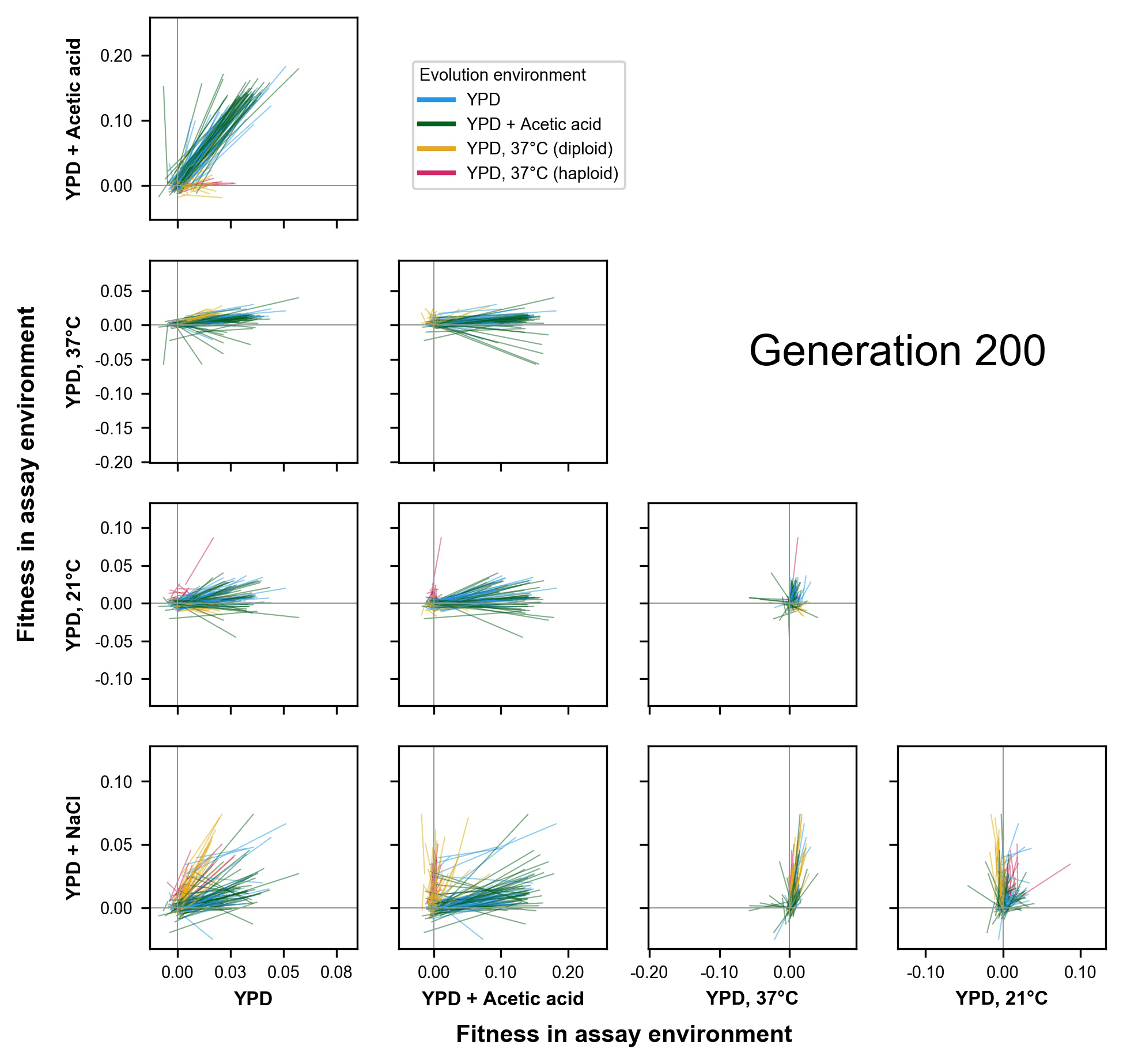

Supplement: Supplementary file 4 [file elife-70918-fig3-video1.gif]
