# Supplementary material for: Dynamics and variability in the pleiotropic effects of adaptation in laboratory budding yeast populations
Source: eLife. 2021 Oct 1;10:e70918. doi: 10.7554/eLife.70918 (PMC8579951; doi:10.7554/eLife.70918)
Supplement: Supplementary file 1. [file elife-70918-supp1.docx]

**SUPPLEMENTARY FILE 1**

*MAT****a*** ancestor

| **Strain** | **Parent** | **Modification** | **Resulting genotype** |
| --- | --- | --- | --- |
| YAN423 | YAN407 | *ycr043c*∆0::*NatMX* | *MAT****a***, *his*3∆1, *ura3*∆0, *leu2*∆0, *lys2*∆0, *RME1*pr::ins-308A, *ycr043c*∆0::*NatMX* |
| YAN434 | YAN423 | *ybr209w*::CORE-UK | *MAT****a***, *his*3∆1, *ura3*∆0, *leu2*∆0, *lys2*∆0, *RME1*pr::ins-308A, *ycr043c*∆0::*NatMX*, *ybr209w*::CORE-UK |
| YAN463 | YAN434 | *can1*::*STE2*pr_*SpHIS5*_*STE3*pr_*LEU2* | *MAT****a***, *his*3∆1, *ura3*∆0, *leu2*∆0, *lys2*∆0, *RME1*pr::ins-308A, *ycr043c*∆0::*NatMX*, *ybr209w*::CORE-UK, *can1*::*STE2*pr_*SpHIS5*_*STE3*pr_*LEU2* |
| YAN467 | YAN463 | *ybr209w*::CORE-UK::*GAL10*pr-*CRE* | *MAT****a***, *his*3∆1, *ura3*∆0, *leu2*∆0, *lys2*∆0, *RME1*pr::ins-308A, *ycr043c*∆0::*NatMX*, *can1*::*STE2*pr_*SpHIS5*_*STE3*pr_*LEU2*, *ybr209w*::*GAL10*pr-*CRE* |
| YCB096A | YAN467 | *TRP1*::GSUK | *MAT****a***, *his*3∆1, *ura3*∆0, *leu2*∆0, *lys2*∆0, *RME1*pr::ins-308A, *ycr043c*∆0::*NatMX*, *can1*::*STE2*pr_*SpHIS5*_*STE3*pr_*LEU2*, *ybr209w*::*GAL10*pr-*CRE*, TRP1::GSUK |
| YCB100A | YCB096A | GSUK::*trp1*∆ | *MAT****a***, *his*3∆1, *ura3*∆0, *leu2*∆0, *lys2*∆0, *RME1*pr::ins-308A, *ycr043c*∆0::*NatMX*, *can1*::*STE2*pr_*SpHIS5*_*STE3*pr_*LEU2*, *ybr209w*::*GAL10*pr-*CRE*, *trp1*∆ |
| YCB123A | YCB100A | *URA3*::*STE5*pr_*URA3* | *MAT****a***, *his*3∆1, *leu2*∆0, *lys2*∆0, *RME1*pr::ins-308A, *ycr043c*∆0::*NatMX*, *can1*::*STE2*pr_*SpHIS5*_*STE3*pr_*LEU2*, *ybr209w*::*GAL10*pr-*CRE*, *trp1*∆, *URA3*::*STE5*pr_*URA3* |
| YCB140B | YCB123A | *HO*::*CgTRP1* | *MAT****a***, *his*3∆1, *leu2*∆0, *lys2*∆0, *RME1*pr::ins-308A, *ycr043c*∆0::*NatMX*, *can1*::*STE2*pr_*SpHIS5*_*STE3*pr_*LEU2*, *ybr209w*::*GAL10*pr-*CRE*, *trp1*∆, *URA3*::*STE5*pr_*URA3*, *HO*::*CgTRP1* |

*MAT****α*** ancestor

| **Strain** | **Parent** | **Modification** | **Resulting genotype** |
| --- | --- | --- | --- |
| YAN424 | YAN404 | *ycr043c*∆0::*HphMX4* | *MAT****α***, *his3*∆1, *ura3*∆0, *leu2*∆0, *lys2*∆0, *RME1*pr::ins-308A, *ycr043c*∆0::*HphMX4* |
| YAN432 | YAN424 | *can1*::*STE2*pr_*SpHIS5*_*STE3*pr_*LEU2* | *MAT****α***, *his3*∆1, *ura3*∆0, *leu2*∆0, *lys2*∆0, *RME1*pr::ins-308A, *ycr043c*∆0::*HphMX4*, *can1*::*STE2*pr_*SpHIS5*_*STE3*pr_*LEU2* |
| YAN446 | YAN432 | *ybr209w*::CORE-UK | *MAT****α***, *his3*∆1, *ura3*∆0, *leu2*∆0, *lys2*∆0, *RME1*pr::ins-308A, *ycr043c*∆0::*HphMX4*, *can1*::*STE2*pr_*SpHIS5*_*STE3*pr_*LEU2*, *ybr209w*::CORE-UK |
| YAN466 | YAN446 | *ybr209w*::CORE-UK::*GAL10*pr-*CRE* | *MAT****α***, *his3*∆1, *ura3*∆0, *leu2*∆0, *lys2*∆0, *RME1*pr::ins-308A, *ycr043c*∆0::*HphMX4*, *can1*::*STE2*pr_*SpHIS5*_*STE3*pr_*LEU2*, *ybr209w*::*GAL10*pr-*CRE* |
| YCB095A | YAN466 | *TRP1*::GSUK | *MAT****α***, *his3*∆1, *ura3*∆0, *leu2*∆0, *lys2*∆0, *RME1*pr::ins-308A, *ycr043c*∆0::*HphMX4*, *can1*::*STE2*pr_*SpHIS5*_*STE3*pr_*LEU2*, *ybr209w*::*GAL10*pr-*CRE*, *TRP1*::GSUK |
| YCB099A | YCB095A | GSUK::*trp1*∆ | *MAT****α***, *his3*∆1, *ura3*∆0, *leu2*∆0, *lys2*∆0, *RME1*pr::ins-308A, *ycr043c*∆0::*HphMX4*, *can1*::*STE2*pr_*SpHIS5*_*STE3*pr_*LEU2*, *ybr209w*::*GAL10*pr-*CRE*, *trp1*∆ |
| YCB122B | YCB099A | *URA3*::*STE5*pr_*URA3* | *MAT****α***, *his3*∆1, *leu2*∆0, *lys2*∆0, *RME1*pr::ins-308A, *ycr043c*∆0::*HphMX4*, *can1*::*STE2*pr_*SpHIS5*_*STE3*pr_*LEU2*, *ybr209w*::*GAL10*pr-*CRE*, *trp1*∆, *URA3*::*STE5*pr_*URA3* |
| YCB137A | YCB122B | *HO*::*CgTRP1* | *MAT****α***, *his3*∆1, *leu2*∆0, *lys2*∆0, *RME1*pr::ins-308A, *ycr043c*∆0::*HphMX4*, *can1*::*STE2*pr_*SpHIS5*_*STE3*pr_*LEU2*, *ybr209w*::*GAL10*pr-*CRE*, *trp1*∆, *URA3*::*STE5*pr_*URA3*, *HO*::*CgTRP1* |
